# Exosomal PD-L1: an effective liquid biopsy target to predict immunotherapy response

**DOI:** 10.1093/nsr/nwy154

**Published:** 2018-12-10

**Authors:** Yanling Song, Lingling Wu, Chaoyong Yang

**Affiliations:** 1 Institute of Molecular Medicine, Renji Hospital, School of Medicine, Shanghai Jiao Tong University, China; 2 MOE Key Laboratory of Spectrochemical Analysis & Instrumentation, Key Laboratory for Chemical Biology of Fujian Province, State Key Laboratory of Physical Chemical of Solid Surfaces, College of Chemistry and Chemical Engineering, Xiamen University, China

Up-regulation of programmed death-ligand 1 (PD-L1) may allow cancer cells to evade the host immune system by recognizing the immune checkpoint programmed death-1 (PD-1) on T cells to promote self-tolerance by suppressing T-cell inflammatory activity [1]. Blocking the interaction between PD-L1 and PD-1 has positive anti-tumor effects [2]. Recently, several antibodies targeting the PD-L1/PD-1 pathway have been approved by the administration for the treatment of various tumors. However, only a minority of patients respond to the therapies [3]. Therefore, a deeper insight into the mechanisms of the immune evasion is urgent to improve the efficacy of these treatments.

**Figure 1.**
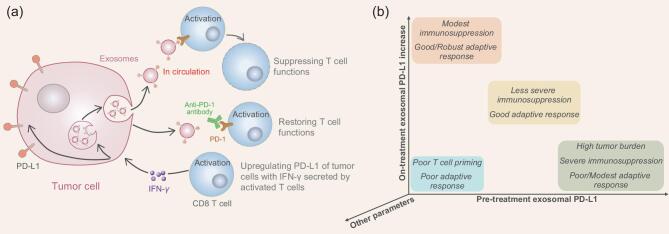
(a) The interactions between circulating exosomal PD-L1 and T cells at different situations. (b) Tracking the levels of circulating exosomal PD-L1 may help to predict patients’ response and identify the possible reasons for success (orange and yellow) or failure (blue and green) for anti-PD-1 therapy.

In recent work by the Guo and Xu groups, they reported that melanoma cells released PD-L1^+^ extracellular vesicles, mostly in the form of exosomes, into the circulation to counter the anti-tumor immunity, unveiling a mechanism by which tumor cells systemically suppress the immune system [4]. The PD-L1 expressed on extracellular vesicles, which predominantly targets PD-1^+^ CD8 T cells, could be up-regulated by interferon-γ (IFN-γ). This finding suggests that PD-L1^+^ extracellular vesicles are able to counteract the immune pressure at the effector stage. In patients with metastatic melanoma before and during anti-PD-1 antibody pembrolizumab treatment, the amount of circulating exosomal PD-L1 may reflect different states of anti-tumor immunity (Fig. 1). Higher levels before treatment may hint at the ‘exhaustion stage’ of T cells where they can hardly be reactivated by the anti-PD-1 therapy. However, a significant increase in the level of circulating exosomal PD-L1 after several weeks of treatment would be a predictor of the adaptive response of the tumor cells to T-cell reinvigoration, stratifying the non-responders in the clinic. The authors found no obvious increment in circulating exosomal PD-L1 during the treatment of non-responders, probably due to the failure of eliciting a sufficient T-cell response or an adaptively down-regulated response to IFN-γ from tumors. The adaptive resistance mechanism is to prevent antigen presentation and also to escape IFN-γ-induced anti-tumor effects. This study investigates in great depth the fundamentals of developing circulating exosomal PD-L1 as an indicator for clinical outcomes and offers possible explanations for the unsatisfactory response rate of anti-PD-1 therapies.

These findings offer novel insights into the tumor-immunosuppression mechanism and provide the rationale for applying exosomal PD-L1 as an effective biomarker for immunotherapy as a complement to tissue biopsy, with the advantages of its non-invasive nature, accurate representation and real-time monitoring, although more basic studies and long-term clinical trials are needed to further confirm the detailed action mechanisms of PD-L1-positive extracellular vesicles and their clinical impact. This work has broadened the scientific community's understanding of PD-L1-mediated tumor immune evasion and the clinical significance of PD-L1 on extracellular vesicles, which would help to develop strategies for accurate monitoring of immunotherapy response and to improve treatment efficacy. In addition to exosomal PD-L1, blood-based tumor mutational burden (bTMB) also can be a clinically actionable biomarker for anti-PD-L1 therapy [5]. It is believed that, in the near future, with further understanding of the immune escape mechanism, there will be more markers or multiple markers combined to predict the immune treatment effect.
